# Neutrophil-to-Lymphocyte Ratio and Platelet-to-Lymphocyte Ratio as Potential Predictors of Prognosis in Acute Ischemic Stroke

**DOI:** 10.3389/fneur.2020.525621

**Published:** 2021-01-25

**Authors:** Cuiping Chen, Lei Gu, Luyun Chen, Wangwang Hu, Xiaowen Feng, Fengzhen Qiu, Zijian Fan, Qitao Chen, Jiayou Qiu, Bei Shao

**Affiliations:** Department of Neurology, The First Affiliated Hospital of Wenzhou Medical University, Wenzhou, China

**Keywords:** acute ischemic stroke, neutrophil-to-lymphocyte ratio, platelet-to-lymphocyte ratio, functional outcomes, predictors

## Abstract

**Objective:** Neutrophil-to-lymphocyte ratio (NLR) and platelet-to-lymphocyte ratio (PLR) have been emerging as the novel inflammatory biomarkers for determining the prognosis of various diseases. This study aimed to investigate the individual and joint effects of NLR and PLR on functional outcomes of acute ischemic stroke (AIS).

**Methods:** Our study involved 448 eligible patients with first-ever AIS. Clinical and laboratory data were collected on admission within 72 h from stroke onset. Unfavorable functional outcome was defined as a modified Rankin Scale score of 3–6 at 3 months after AIS. Cox proportional hazard model and spline regression models was used to estimate the effect of NLR and PLR on risk of adverse outcomes after the last patient who completed a 3-months follow-up was enrolled.

**Results:** After adjusting confounders, NLR were significantly associated with the unfavorable functional outcomes (*P*-trend < 0.001). So were PLR (*P*-trend < 0.001). NLR was discovered to have higher predictive value than PLR (AUC = 0.776, 95%CI = 0.727–0.825, *P* < 0.001; AUC = 0.697, 95%CI = 0.641–0.753, *P* < 0.001). The optimal cutoff values for NLR and PLR was 3.51 and 141.52, respectively. Stratified analysis performed by cox proportional hazard model showed that high level of NLR and PLR (NLR ≥ 3.51, PLR ≥ 141.52) presented the highest risk of unfavorable functional outcomes (adjusted HR, 3.77; 95% CI: 2.38–5.95; *P* < 0.001). Followed by single high level of NLR (adjusted HR, 2.32; 95% CI: 1.10–4.87; *P* = 0.027). Single high level of PLR (NLR < 3.51, PLR ≥ 141.52) also showed higher risk than low level of the combination, but it did not reach statistical significance (adjusted HR, 1.42; 95% CI: 0.75–2.70; *P* = 0.285). No obvious additive [relative excess risk due to interaction (RERI) not significant] or multiplicative (adjusted HR, 0.71; 95%CI: 0.46–1.09; *P* = 0.114) interaction was found between the effects of NLR and PLR on the risk of unfavorable functional outcomes.

**Conclusion:** This study demonstrated that both NLR and PLR were independent predictors of 3-months functional outcomes of AIS. They may help to identify high-risk patients more forcefully when combined together.

## Introduction

Stroke, the second leading cause of deaths and the third main malady giving rise to disability worldwide, has brought about a major drain on public health-care funding (1). The most common subtype of stroke is ischemic stroke, accounting for 80% of all (2). As China has the highest number of stroke cases in the world with its incidence and prevalence escalating and sprawling over the past decade (3), long-term care of the patients laid a immeasurably huge burden on thousands relatives of patients and caregivers. For this reason, identifying biomarkers for predicting ischemic stroke and accurately evaluating its prognosis is salutary and truly preoccupying to the families.

Post-ischemic inflammation plays an important role in various stages of cerebral ischemic injury, resulting from stagnant blood flow, activation of intravascular leukocytes and release of pro-inflammatory mediators from the ischemic endothelium, platelet granules, and brain parenchyma (4, 5). Numerous studies confirmed that inflammatory response aggravated ischemic brain damage and neurological dysfunction (4, 6, 7). Previous researches suggested that leukocytosis on admission was associated with stroke severity and poor clinical outcomes in acute ischemic stroke (AIS) patients (8). High neutrophil count and low lymphocyte count were regarded as correlation factors of unfavorable functional outcomes of acute cerebral infarction (9). Although the relativity between an increasing platelet count and clinical prognosis remained to be uncertain, it was confirmed that platelets acted as a pivotal role in thrombogenesis and inflammation (5, 10).

Neutrophil-to-lymphocyte ratio (NLR) and platelet-to-lymphocyte ratio (PLR) as newfound and inexpensive biomarkers in systematic inflammation were proved to possess diagnostic and predictive capabilities in multiple diseases recently (11–13). Their application in cerebrovascular diseases have been spotlighted by many. Xue et al. found that NLR in patients with AIS was related to stroke severity, short-term functional prognosis and recurrence of cerebral infarction (14). Kocaturk et al. believed that NLR could portend short-term mortality in patients with AIS (15). Altintas et al. elucidated that high PLR augmented the infarct volume and the incidence of undesirable prognosis in AIS patients during 3-years follow up (16). Lately, The joint application of NLR and PLR for predicting clinical prognosis has been universally applied to patients with cancer, coronary artery disease, and subarachnoid hemorrhage (17–19). However, to the best of our knowledge, there has been no data supporting the significance of the joint effects of NLR and PLR on short-term functional outcomes of AIS. Therefore, the aim of this study was to investigate the individual and joint prognostic value of NLR and PLR in acute ischemic stroke (AIS).

## Methods

### Study Population

From Oct. 2017 to Sep. 2018, the patients who suffered first-ever AIS and visited the First Affiliated Hospital of Wenzhou Medical University within 72 h from symptom onset were consecutively recruited in this prospective study. All the patients were newly diagnosed with AIS in accordance with clinical symptoms and neuroimages listed in the World Health Organization criteria (20). This study was approved by the ethics committee of the First Affiliated Hospital of Wenzhou Medical University. All the patients or their relatives signed the consent forms before inclusion.

Exclusion criteria for the patients was as follows: (1) thrombolysis therapy and mechanical endovascular therapy; (2) clinical or laboratory findings in conformity with the presence of acute or chronic infection (e.g., pneumonia, urinary tract infection, inflammation in other sites); (3) surgery, severe trauma or burn within 90 days before the symptom onset; (4) hematologic disorders, rheumatoid immune-related diseases, or malignancy; (5) use of steroids or immunosuppressive agents; (6) severe liver and kidney dysfunction.

### Data Collection

The following baseline clinical information was obtained within 72 h of admission, comprising age, gender, systolic blood pressure, diastolic blood pressure and the history of smoking, alcohol drinking, hypertension, diabetes mellitus, hyperlipidemia, atrial fibrillation, and medications. Hypertension was defined as repeated multiple systolic blood pressure ≥ 140 mmHg and (or) diastolic blood pressure ≥ 90 mmHg by a previously established diagnosis or the utilization of antihypertensive medicines. Diabetes mellitus was determined as a fasting plasma glucose of ≥7 mmol/L, a random plasma glucose of ≥11.1 mmol/L, or HbA1c of ≥6.5%, and the history of diagnosed diabetes or use of diabetic medications. Hyperlipidemia was confirmed as a high level of serum total cholesterol (≥5.2 mmol/L), triglycerides (≥1.7 mmol/L), low-density lipoprotein cholesterol (≥3.4 mmol/L), and high-density lipoprotein (<1.0 mmol/L) or previous diagnosis of hyperlipidemia. Atrial fibrillation (AF) was diagnosed according to conclusive electrocardiogram data or any previously known AF episode. Smoking was identified as taking more than 1 cigarette per day for 6 months. Alcohol drinking was recognized as the average drinking of 2 U/d for male or 1 U/d for female. Medications including the use of antiplatelet agents, anticoagulants or statins were recorded at the time of discharge.

Complete blood cell count consisting of total white blood cell count, neutrophil count, lymphocyte count and platelet count, were collected at the time of admission. NLR and PLR were calculated as the ratio of neutrophil to lymphocyte count and that of platelet to lymphocyte count, respectively. Other laboratory parameters such as total cholesterol, triglycerides, low-density lipoprotein cholesterol, high-density lipoproteins, serum creatinine, blood urea nitrogen, uric acid, homocysteine, fasting blood glucose, postprandial blood glucose and glycated hemoglobin were detected from the blood samples of the patients extracted on the first morning after admission. All the items were tested in the department of biochemistry of our hospital.

Severity of ischemic stroke was measured on admission using the National Institutes of Health Stroke Scale (NIHSS) (21). With reference to other previous study, we defined mild stroke as NIHSS scores below 6, while moderate to severe stroke as NIHSS scores over or tantamount to 6 (22). The etiologic subtypes of ischemic stroke were categorized by large-artery atherosclerosis, cardioembolism, small-vessel diseases, or other determined etiology and undetermined etiology according to the Trial of Org 10172 in Acute Stroke Treatment (TOAST) criteria (23).

Patients or their family members were followed up by telephone interviews or outpatient department of our hospital by two experienced neurologists. Functional outcomes of AIS patients after 3 months were evaluated using the modified Rankin Scale (mRS, scores ranged from 0 to 6) (24). The follow-up was terminated at the expiration of 3 months after AIS or at the occurrence of deceases. An unfavorable functional outcome was defined as mRS ≥ 3, and a favorable functional outcome as mRS < 3 (25).

### Statistical Analysis

Statistical analyses were performed by the SPSS version 22.0 (SPSS Inc., Chicago, IL). Normality of distribution was analyzed by the Kolmogorov–Smirnov test. Categorical variables expressed as counts and percentages were calculated using Chi-square test. Continuous variables were depicted by means and standard deviations (SD) or medians and interquartile ranges (IQR) depending on the normality of data distribution, and assessed by two independent samples *t*-test or Mann-Whitney *U*-test as appropriate. Variables with *P*-value below 0.1 on univariate analysis were selected as covariates. NLR were divided into quartiles (Q1, <1.80; Q2, ≥1.80 and <2.62 mmol/L; Q3, ≥2.64 and <4.06 mmol/L and Q4, ≥4.06 mmol/L) and PLR were also were categorized (Q1, <103.20; Q2, ≥103.21 and <134.29 mmol/L; Q3, ≥134.91 and <185.66 mmol/L and Q4, ≥186.02 mmol/L). Cox proportional hazard model and spline regression models was used to estimate the effect of NLR and PLR on risk of unfavorable outcomes. Receiver operating characteristic (ROC) curves were adopted to evaluate the accuracy of NLR and PLR in predicting 3-months functional outcomes and determine the cut-point thresholds. According to the optimal cut-off values, all the patients were divided into four groups. Then stratify analysis was proceeded by cox proportional hazard model. The interaction between NLR and PLR was analyzed. A 2-sided *P* < 0.05 was considered to be statistically significant.

## Results

**Figure 1:**
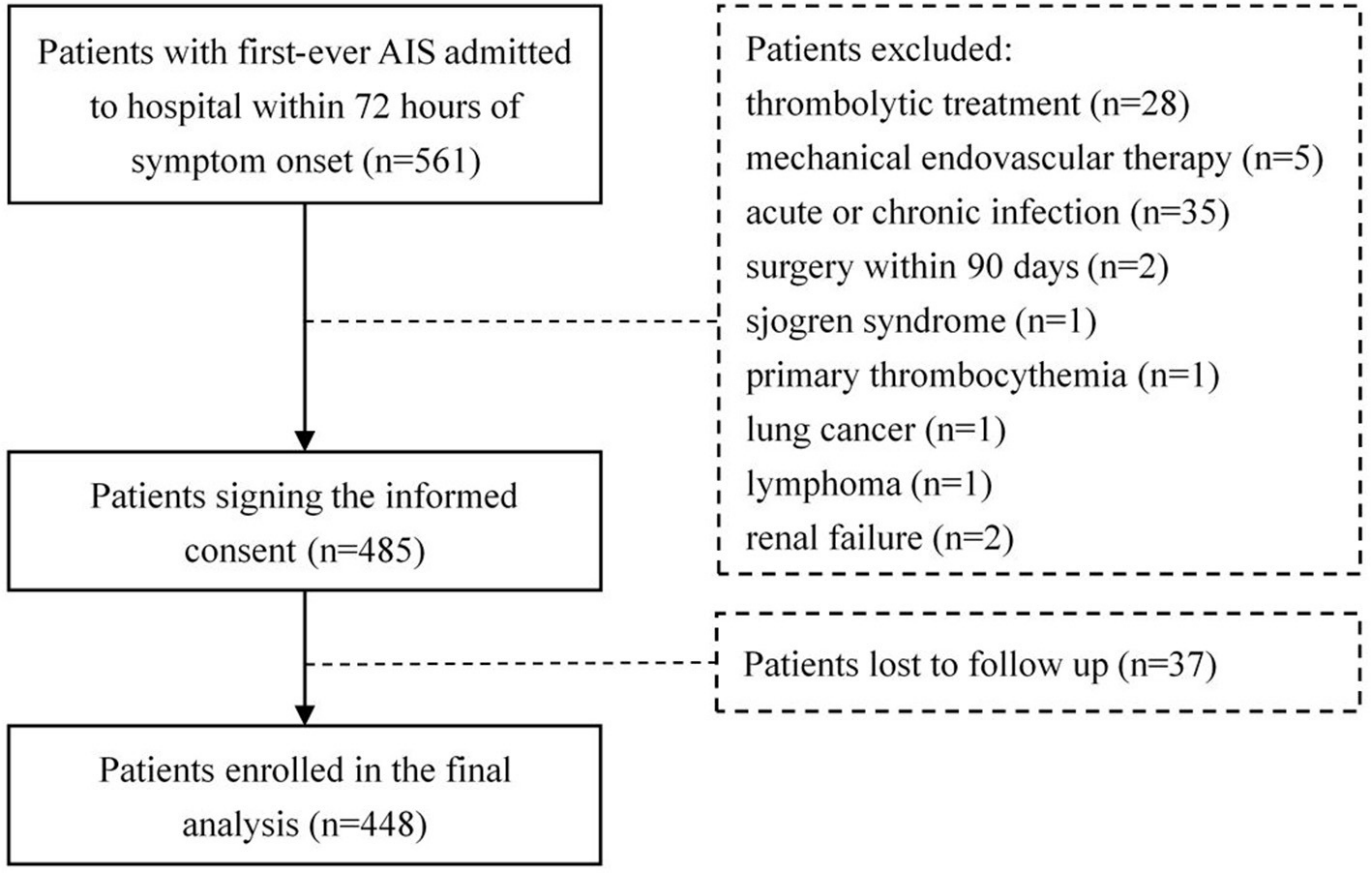
Research flowchart showing the patient selection.

As a result, 561 patients with newly diagnosed AIS were admitted to the hospital within 72 h from symptom onset. Of these patients, 76 meeting the exclusion criteria were ruled out, 37 patients withdrew from the study during the follow-up. Finally, 448 eligible patients, including 290 males (64.7%), were enrolled in our study (Figure 1). A total of 37 among these cases deceased during follow-up with a median follow-up time of 1.2 months (interquartile range 0.6–1.5).

Baseline characteristics of all the participants were shown in Table 1. The median (quartiles) NIHSS score at admission was 4 (IQR 2–5) and unfavorable outcomes at 3-months was found in 130 patients (29.0%). The average age was 66.8 ± 12.2 years old. Compared with the favorable prognosis group, patients with unfavorable outcome were significantly older, with higher NIHSS scores on admission, more occurrence of hypertension and AF and more usage count of anticoagulants (all *P* < 0.05); the laboratory figures of white blood cell count, neutrophil count, platelet count, NLR, PLR and blood urea nitrogen were higher in the unfavorable outcome group, while lymphocyte count was significantly lower (all *P* < 0.05). Moreover, the distribution of etiological classification between the two groups was significantly distinct (*P* = 0.001). Other baseline data between two groups presented non-significant difference (*P* > 0.05).

**Table 1:** Baseline characteristics of AIS patients with favorable or unfavorable outcomes.

**Characteristics**	**Total**	**The prognosis of 3 months**		
		**Favorable outcomes (*n* = 318)**	**Unfavorable outcomes (*n* = 130)**	***P*-value**
Age (years)	66.8 ± 12.2	64.9 ± 12.1	71.5 ± 11.0	<0.001^a^
Male (*n*)	290 (64.7)	211 (66.4)	79 (60.8)	0.262^c^
SBP (mmHg)	153.7 ± 22.8	153.2 ± 23.4	154.9 ± 21.4	0.469^a^
DBP (mmHg)	82.0 ± 12.8	82.3 ± 13.1	81.2 ± 12.1	0.433^a^
Admission to hospital (d)	2.0 (1.0–3.0)	2.0 (1.0–3.0)	2.0 (1.0–3.0)	0.925^b^
Smoking (*n*)	193 (43.1)	146 (45.9)	57 (48.9)	0.690^c^
Alcohol drinking (*n*)	127 (28.3)	98 (30.8)	29 (22.3)	0.145^c^
NIHSS score on admission	4.0 (2.0–5.0)	3.0 (1.0–4.0)	7.0 (4.0–10.0)	<0.001^b^
**Comorbidities**				
Hypertension (*n*)	367 (81.9)	253 (79.6)	114 (87.7)	0.042^c^
Diabetes (*n*)	172 (38.4)	119 (37.4)	53 (40.8)	0.508^c^
Dyslipidemia (*n*)	174 (38.8)	131 (41.2)	43 (33.1)	0.110^c^
Atrial fibrillation (*n*)	53 (11.8)	27 (8.5)	26 (20.0)	0.001^c^
Stroke etiologic subtypes				0.001^c^
Large-artery atherosclerosis (*n*)	292 (65.2)	209 (65.7)	83 (63.9)	
Cardioembolic (*n*)	57 (12.7)	31 (9.8)	26 (20.0)	
Small-vessel disease (*n*)	53 (11.8)	47 (14.8)	6 (4.6)	
Other or unknown cause (*n*)	46 (10.3)	31 (9.8)	15 (11.5)	
**Laboratory findings**				
WBC (109/L)	6.9 (5.7–8.2)	6.6 (5.5–7.8)	7.6 (6.2–9.3)	<0.001^b^
Neutrophils (109/L)	4.3 (3.3–5.7)	4.0 (3.2–4.9)	5.6 (4.3–7.2)	<0.001^b^
Lymphocytes (109/L)	1.7 ± 0.7	1.8 ± 0.7	1.4 ± 0.6	<0.001^a^
Platelets (109/L)	225.1 ± 65.3	221.3 ± 57.4	234.6 ± 81.0	0.090^a^
NLR	2.6 (1.8–4.1)	2.3 (1.7–3.2)	4.3 (2.9–6.2)	<0.001^b^
PLR	134.6 (103.2–185.9)	123.4 (96.4–166.3)	173.8 (126.1–242.7)	<0.001^b^
TC (mmol/L)	4.4 (3.7–5.1)	4.5 (3.8–5.3)	4.4 (3.6–4.9)	0.134^b^
TG (mmol/L)	1.5 (1.2–2.1)	1.6 (1.2–2.3)	1.5 (1.1–1.8)	0.127^b^
HDL (mmol/L)	1.0 (0.9–1.2)	1.0 (0.9–1.2)	1.0 (0.9–1.2)	0.800^b^
LDL (mmol/L)	2.6 ± 0.8	2.6 ± 0.8	2.5 ± 0.7	0.128^a^
SCr (μmol/L)	71.0 (59.0–84.5)	71.0 (58.0–85.0)	70.0 (59.3–84.0)	0.914^b^
BUN (mmol/L)	5.1 (4.1–6.4)	4.9 (4.1–6.1)	5.7 (4.3–7.3)	<0.001^b^
UA (mmol/L)	290.5 (232.5–352.0)	294.0 (240.5–352.5)	282.0 (208.0–352.0)	0.256^b^
HCY (mmol/L)	11.0 (9.0–14.0)	11.0 (9.0–13.0)	11.5 (8.0–15.0)	0.383^b^
FBG (mmol/L)	5.3 (4.6–6.8)	5.2 (4.5–6.7)	5.4 (4.7–7.1)	0.083^b^
PBG (mmol/L)	8.1 (6.3–11.5)	8.2 (6.4–11.7)	8.0 (6.3–11.3)	0.790^b^
HbA1c (%)	6.0 (5.6–7.1)	6.0 (5.6–7.3)	6.0 (5.5–6.6)	0.800^b^
**Medications**				
Antiplatelet agents (*n*)	341 (76.1)	248 (78.0)	93 (71.5)	0.146^c^
Anticoagulation agents (*n*)	40 (8.9)	19 (6.0)	21 (16.2)	0.010^c^
Statin (*n*)	349 (77.9)	245 (77.0)	104 (80.0)	0.494^c^

*SBP, systolic blood pressure; DBP, diastolic blood pressure; WBC, white blood cell; NLR, neutrophil-to-lymphocyte ratio; PLR, platelet-to-lymphocyte ratio; TC, total cholesterol; TG, triglycerides; HDL, high-density lipoprotein; LDL, low density lipoprotein; SCr, serum creatinine; BUN, blood urea nitrogen; UA, uric acid; HCY, homocysteine; FBG, fasting blood glucose; PBG, postprandial blood glucose; HbA1C, glycated hemoglobin*.

*Data are presented as means ± standard deviations and medians (interquartile ranges) or as counts (percentages)*.

a *Two independent samples t-test*.

b *Mann-Whitney U-test*.

c *Chi-square test*.

**Figure 2:**
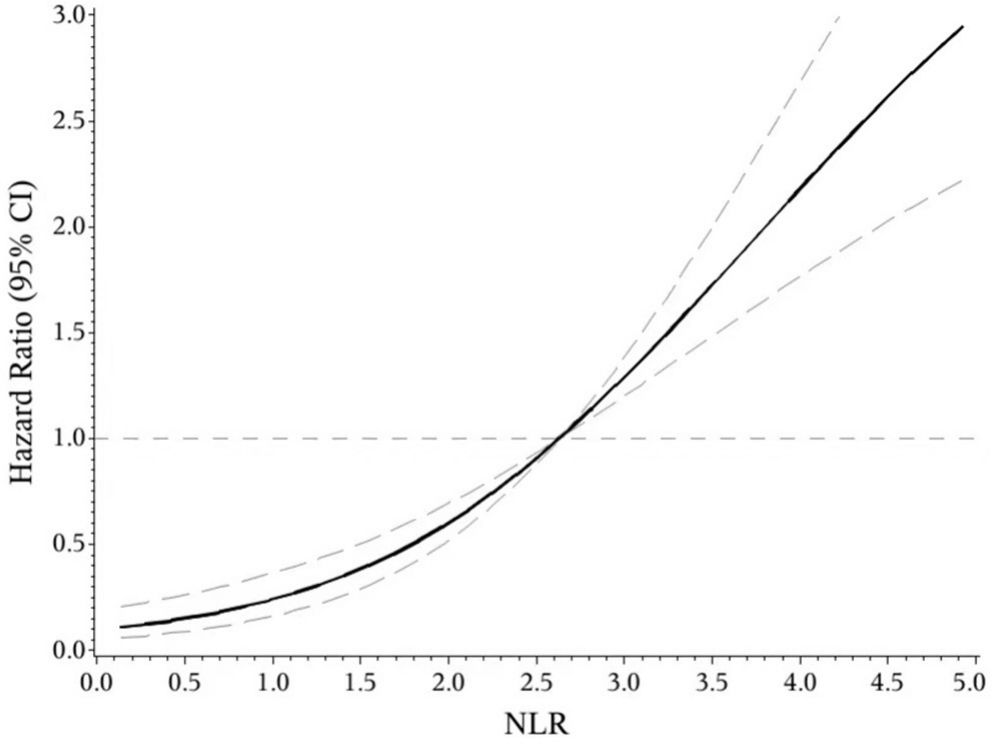
ROC curves of NLR and PLR for predicting 3-months functional outcomes in acute ischemic stroke.

**Figure 3:**
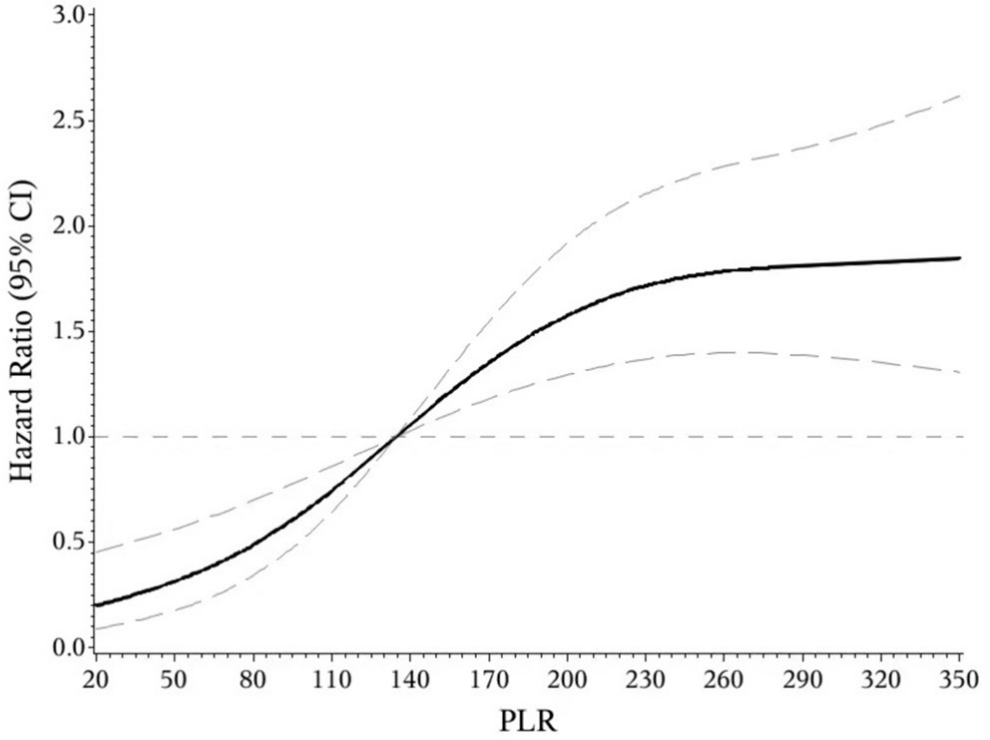
Association of NLR level to the risk of poor functional outcomes based on spline regression model.

The indicators that differed significantly with *P* < 0.1 in univariate analysis comprising age, hypertension, atrial fibrillation, NIHSS score on admission, etiologic subtypes of stroke, fasting blood glucose and blood urea nitrogen were incorporated into cox proportional hazard model, determining the independent effect of individual NLR and PLR on clinical prognosis. After adjusting confounders, NLR were significantly associated with the unfavorable functional outcomes (adjusted HR for Q3 vs. Q1, 3.475; 95% CI: 1.579–7.648; adjusted HR for Q4 vs. Q1, 6.121; 95% CI: 2.887–12.977; *P*-trend < 0.001). So were PLR (adjusted HR for Q3 vs. Q1, 2.42; 95% CI: 1.30–4.49; adjusted HR for Q4 vs. Q1, 3.54; 95% CI: 1.91–6.54; *P*-trend < 0.001) (Table 2). Spline regression showed a dose–response relationship between NLR, PLR levels with risk of unfavorable outcomes (Figures 2, 3).

**Table 2:** Risk of unfavorable outcomes of participants associated with NLR and PLR.

**Variables**	***n***	**Unfavorable outcomes, # (%)**	**Crude**		**Adjusted**	
			**HR (95% CI)**	***P*-value**	**HR (95% CI)**	***P*-value**
Per SD increment of NLR	448	130 (29.02%)	1.72 (1.47, 2.00)	<0.001	1.51 (1.27, 1.80)	<0.001
**Quartile**						
Q1 (<1.80)	112	9 (8.04)	1.00 (1.00, 1.00)	Ref.	1.00 (1.00, 1.00)	Ref.
Q2 (1.80 2.62)	112	18 (16.07)	1.96 (0.88, 4.36)	0.010	1.710 (0.732, 3.996)	0.215
Q3 (2.64 4.06)	112	32 (28.57)	3.57 (1.71, 7.49)	0.001	3.475 (1.579, 7.648)	0.002
Q4 (≥4.06)	112	71 (63.39)	8.34 (4.17, 16.70)	<0.001	6.121 (2.887, 12.977)	<0.001
P for trend				<0.001		<0.001
Per SD increment of PLR	448	130 (29.02%)	1.63 (1.35, 1.95)	<0.001	1.61 (1.29, 2.00)	<0.001
Q1 (<103.20)	112	17 (15.18)	1.00 (1.00, 1.00)	Ref.	1.00 (1.00, 1.00)	Ref.
Q2 (103.21 134.29)	112	20 (17.86)	1.15 (0.60, 2.20)	0.674	1.47 (0.74, 2.91)	0.270
Q3 (134.91 185.66)	112	35 (31.25)	1.85 (1.04, 3.30)	0.038	2.42 (1.30, 4.49)	0.005
Q4 (≥186.02)	112	58 (51.79)	3.18 (1.85, 5.47)	<0.001	3.54 (1.91, 6.54)	<0.001
P for trend				<0.001		<0.001

*Adjusted for age, hypertension, atrial fibrillation, NIHSS score on admission, stroke etiologic subtypes, FBG, BUN, anticoagulation agents*.

**Figure 4:**
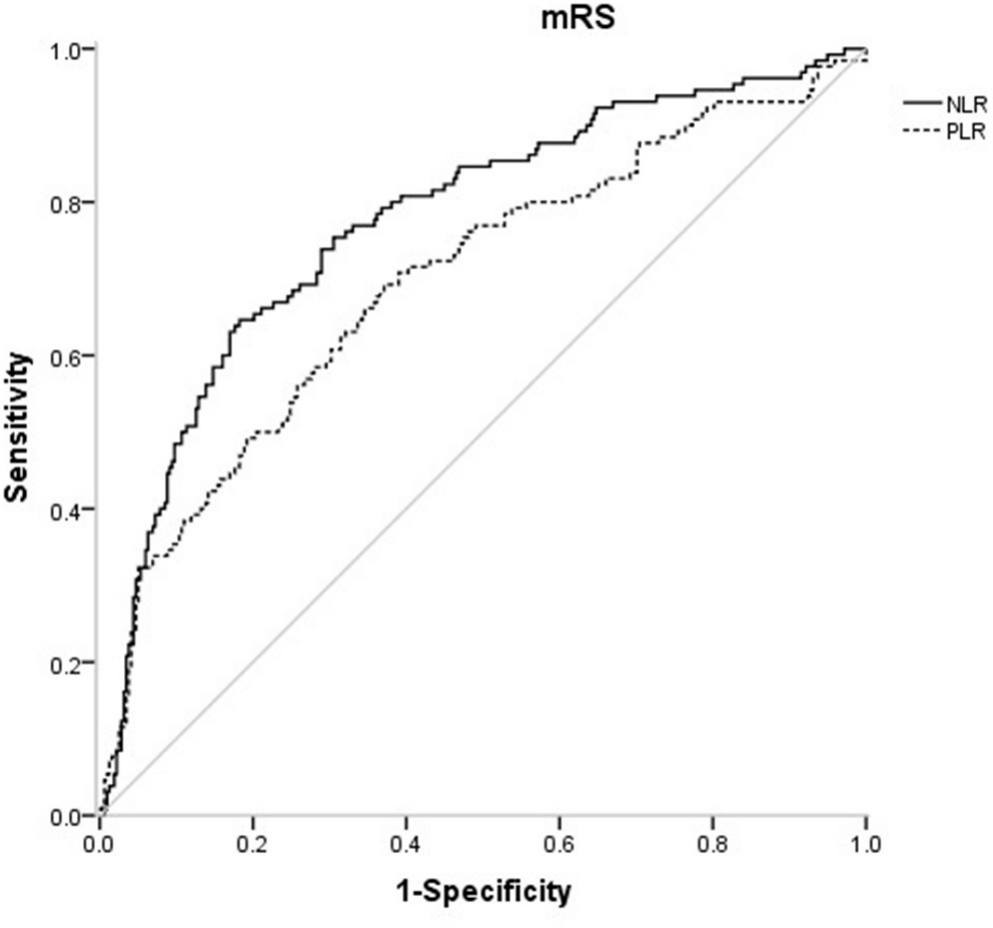
Association of PLR level to the risk of poor functional outcomes based on spline regression model.

In the receiver operating characteristic (ROC) curve analysis, we identified the optimal cut-off value of NLR as 3.51 (with sensitivity of 64.6% and specificity of 81.8%) for the unfavorable outcomes with area under curve (AUC) of 0.776 (95%CI = 0.727–0.825, *P* < 0.001). The optimal cut-off value of PLR for 3-months unfavorable prognosis was 141.52 with sensitivity of 69.2% and specificity of 62.9% (AUC, 0.697; 95%CI = 0.641–0.753, *P* < 0.001). NLR presented higher predictive value than PLR in 3-months functional outcomes of AIS (0.078 7, 95%CI = 0.034 9–0.122, *P* = 0.000 4) (Figure 4).

According to the optimal cut-off values, all the patients were divided into four groups (NLR ≥ 3.51, PLR ≥ 141.52; NLR ≥ 3.51, PLR < 141.52; NLR < 3.51, PLR ≥ 141.52; NLR < 3.51, PLR < 141.52) (Table 3). Stratified analysis performed by cox proportional hazard model showed that high level of NLR and PLR (NLR ≥ 3.51, PLR ≥ 141.52) presented the highest risk of unfavorable functional outcomes (adjusted HR, 3.77; 95% CI: 2.38–5.95; *P* < 0.001). Followed by single high level of NLR (adjusted HR, 2.32; 95% CI: 1.10–4.87; *P* = 0.027). Single high level of PLR (NLR < 3.51, PLR ≥ 141.52) also showed higher risk than low level of the combination, but it did not reach statistical significance (adjusted HR, 1.42; 95% CI: 0.75–2.70; *P* = 0.285). No obvious additive [relative excess risk due to interaction (RERI) and the synergy index S were not significant] or multiplicative (adjusted HR, 0.71; 95%CI: 0.46–1.09; *P* = 0.114) interaction was found between the effects of NLR and PLR on the risk of unfavorable functional outcomes.

**Table 3:** Joint effects of NLR and PLR on unfavorable outcomes related acute ischemic stroke.

**NLR≥3.51**	**PLR≥141.52**	***N***	**Unfavorable outcomes, # (%)**	**Crude**		**Adjusted**	
				**HR (95% CI)**	***P*-value**	**HR (95% CI)**	***P*-value**
Yes	Yes	117	74 (63.25)	Ref	Ref	3.77 (2.38–5.95)	<0.001
Yes	No	25	10 (40.00)	0.61 (0.32, 1.18)	0.141	2.32 (1.10–4.87)	0.027
No	Yes	91	16 (17.58)	0.26 (0.15, 0.44)	<0.001	1.42 (0.75–2.70)	0.285
No	No	215	30 (13.95)	0.22 (0.15, 0.34)	<0.001	Ref	Ref
Interaction of NLR and PLR				1.43 (0.58, 3.50)	0.436	0.71 (0.46, 1.09)	0.114
RERI (95% CI) = 0.27 (−0.22, 0.77)							
The synergy index S = 0.73 (0.45, 1.19)							

*Adjusted for age, hypertension, atrial fibrillation, NIHSS score on admission, stroke etiologic subtypes, FBG, BUN, anticoagulation agents*.

## Discussion

This prospective and observational study came out with NLR and PLR as the independent predictors for functional outcomes of AIS after 3 months, which further confirms previous studies (14, 16). On this basis, the present study may be the first one to provide quantified evidence between NLR, PLR and risk of short-term unfavorable functional outcomes. We found a J-shaped relation between NLR and higher risk of poor functional outcomes, an S-shaped relation between PLR and poor functional outcomes in ischemic stroke patients. In addition, as current researches over joint effect of NLR and PLR on the prognosis of AIS were limited (26), we found that the combined application of NLR and PLR can more effectively identify high-risk patients, which is our innovation.

There has been increasing evidence suggests that post-ischemic inflammation plays a critical role in ischemia stroke. On the occurrence of cerebral ischemia, proteins such as brain-derived antigens, danger-associated molecular patterns (DAMPs), cytokines and chemokines were released from the injured brain regions into the systemic circulation (27–29). Many of these factors triggered a series of systemic pro-inflammatory molecules and cellular events that exacerbated brain damage in acute and late clinical outcomes (27, 30). The climbing level of white blood cell count at the early stage of AIS was proven to be related to a larger stroke volume, more severe stroke deficits and negative clinical outcomes (8, 31). In the white blood cell family, neutrophils were the first to infiltrate the ischemic brain (taking focal cerebral ischemia for instance, the permeation time ranged from 30 min to several hours) and reached the peak early (1–3 days post stroke) (32). Neutrophils deteriorated the injured brain by direct neurotoxic effects of releasing proteolytic enzymes (33). A SPECT study by Price et al. demonstrated the accumulation of radio-labeled neutrophils related to infarct severity and growth and pessimistic neurologic outcomes (34). Lymphocytes were suggested to be a pivotal subtype that determined the severity of neuroinflammation with complicated and diverse influences in acute brain injuries. So were other pro-inflammatory lymphocytes involving TH1, TH17, and γδ T-cells verified by several researches (35, 36). Oppositely, regulatory T-cells (Treg) and B-cells (Breg) shown the potential of neuroprotective effects (37). In Kim's study, the effects of lymphocytes on prognosis of stroke were investigated with findings that lower lymphocyte count were associated with 3-months unfavorable functional outcomes (9). The time course of the recruitment of lymphocyte-subtypes into the ischemic brain regions remained unclear. Earlier studies indicated that lymphocytes assembled in the lesion at the late stages of ischemic brain injury, whereas more recent studies in rodent models demonstrated an early accumulation of T cells in the injured regions within the first 24 h from stroke onset (38). Circulating platelets presented two major functions in ischemic stroke: Firstly, directly resulting in the formation of circulating arterial thrombosis and embolism (10); secondly, acting as a prime motor of the activators stored in platelet granules (e.g., chemokines and cytokines) that mediated other peripheral blood cells (5). D'Erasmo et al. indicated that platelet count was significantly lower in patients with ischemic stroke compared with healthy controls (39). Du et al. suggested that boosted platelet count risen the risk of ischemic stroke yet without significant association with poor prognosis (40). Recently, Yang et al. discovered that platelet count may be a qualified predictor for recurrent stroke, mortality, and poor functional outcome in ischemic stroke or TIA patients with platelet count within normal range (41). In our study, unfavorable functional outcome group owned higher neutrophil count but lower lymphocyte count than the favorable functional outcome group. However, the platelet count was non-significantly higher in patients with unfavorable outcomes than the favorable ones.

**Figure 5:**
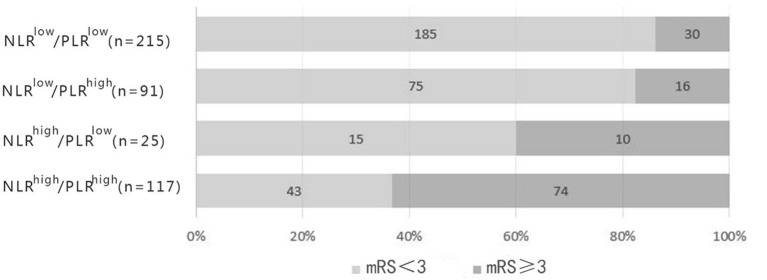
Distribution of 3-months functional outcomes of groups.

As the passages have cleared, NLR and PLR are repeatable and novel composite biomarkers to determine the severity of the systemic inflammatory burden in ischemic stroke (11–13). They retained respective prognostic values as a single form and a mixed form with two prominent features as follows: First, the combined index reflected pro-inflammatory or pro-coagulant status and immunosuppression, resulting in precise selection of common biomarkers; second, the ratios were more stable than a single blood parameter that could be impacted by several variables, such as dehydration, over-hydration and blood specimen handling (42). Our findings that NLR and PLR correlated with clinical outcomes in AIS patients were consistent with the previous studies, underpinning the significance of NLR and PLR in the foreknowledge of pathological courses of AIS. The different predictability of NLR and PLR for AIS in our study broadened the choices of manageable biomarkers to quench the risk factors at the early stage of stroke. In our study, high level of NLR and PLR (NLR ≥ 3.51, PLR ≥ 141.52) presented the highest risk of unfavorable functional outcomes. As shown in Figure 5, the distribution of 3-months mRS outcomes of NLR^high^/PLR^high^ were substantially shifted to worse outcomes. The possible explanation may be due to the different subtle effects of NLR and PLR. The former mainly represents inflammatory damage, while the latter has thrombosis in addition to inflammation. This may help us more effectively identify high-risk patients.

Strengths of this study include the prospective desigh, the quantitative relationship between indicators with outcomes and the conjoint analysis of two indicators. However, our study existed several limitations that need expounding. First, our study was a single-center study dearth of large sample size and strong representation of the population, resulting in a slight selection bias. Second, this study precluded the AIS patients undergoing thrombolysis therapy and mechanical endovascular therapy. We ruminated the desirability of these patients who should be involved in the future. Third, our study did not determine the rehabilitation of patients after discharge, which is still an important part of functional recovery after stroke. Fourth, the blood samples were only collected within 72 h from symptom onset. Without serial measurements, so the association between the dynamic change of NLR and PLR ratio and the prognosis in AIS patients remains unclear.

## Conclusion

In conclusion, our study suggested that both NLR and PLR could be promising indicators for predicting the functional prognosis of AIS. We found a J-shaped relation between NLR and higher risk of poor functional outcomes, an S-shaped relation between PLR and poor functional outcomes in ischemic stroke patients. They may help to identify high-risk patients more powerfully when combined together. More supportive evidence was required to guide the clinical application of NLR and PLR.

## Data Availability Statement

All datasets generated for this study are included in the article/supplementary material.

## Ethics Statement

The studies involving human participants were reviewed and approved by Ethics Committee of the First Affiliated Hospital of Wenzhou Medical University. The patients/participants provided their written informed consent to participate in this study. Written informed consent was obtained from the individual(s) for the publication of any potentially identifiable images or data included in this article.

## Author Contributions

CC: data curation and writing—original draft. LG: analyze statistics and plot. LC, WH, XF, FQ, ZF, QC, and JQ: investigation and resources. All authors contributed to the article and approved the submitted version.

## Conflict of Interest

The authors declare that the research was conducted in the absence of any commercial or financial relationships that could be construed as a potential conflict of interest.
